# An Art and Industrial Exhibition in the Capitol at Washington, D. C.

**Published:** 1882-09-20

**Authors:** 


### *AN ART AND INDUSTRIAL EXHIBITION IN THE CAPITOL AT WASHINGTON,*

*Under the Auspices of the Society of the Army of the Cumberland, for the Benefit of the Garfield Monument Fund.*

A National Bazaar, Art, and Industrial Exposition will be held in the rotunda and adjacent halls of the National Capitol at Washington, D. C., November 25th to December 3d, (inclusive), 1882, as authorized by joint resolution of the Senate and House of Representatives, August 7, 1882. The object of this undertaking is to raise funds with which to erect a statue in this city to the memory of Gen. JAMES A. GARFIELD, late President of the United States, which work is in the hands of a committee of the Society of the Army of the Cumberland, who have already collected for this purpose some twenty thousand dollars, and expect, with the results of the Exposition, to have a sufficient sum with which to erect a work befitting the great name it is proposed to commemorate.

